# Identification of TMEM208 and PQLC2 as reference genes for normalizing mRNA expression in colorectal cancer treated with aspirin

**DOI:** 10.18632/oncotarget.15191

**Published:** 2017-02-08

**Authors:** Yuanyuan Zhu, Chao Yang, Mingjiao Weng, Yan Zhang, Chunhui Yang, Yinji Jin, Weiwei Yang, Yan He, Yiqi Wu, Yuhua Zhang, Guangyu Wang, Riju James RajkumarEzakiel Redpath, Lei Zhang, Xiaoming Jin, Ying Liu, Yuchun Sun, Ning Ning, Yu Qiao, Fengmin Zhang, Zhiwei Li, Tianzhen Wang, Yanqiao Zhang, Xiaobo Li

**Affiliations:** ^1^ Department of Gastrointestinal Medical Oncology, The Affiliated Tumor Hospital of Harbin Medical University, Harbin, China; ^2^ Department of Pathology, Harbin Medical University, Harbin, China; ^3^ Department of Nutrition and Food Hygiene, Public Health College, Harbin Medical University, Harbin, China; ^4^ Department of Sports Humanities, Harbin Sport University, Harbin, China; ^5^ Center of Digital Dentistry, Pecking University School and Hospital of Stomatology, Beijing, China; ^6^ Department of Gastrointestinal Surgery, International Hospital of Pecking University, Beijing, China; ^7^ Department of Histology and Embryology, Harbin Medical University, Harbin, China; ^8^ Department of Microbiology, Harbin Medical University, Harbin, China; ^9^ The Northern Medicine Translational Center, Heilongjiang Province Academy of Medical Science, Harbin, China

**Keywords:** aspirin, reference gene, colorectal cancer

## Abstract

Numerous evidences indicate that aspirin usage causes a significant reduction in colorectal cancer. However, the molecular mechanisms about aspirin preventing colon cancer are largely unknown. Quantitative reverse transcription polymerase chain reaction (qRT-PCR) is a most frequently used method to identify the target molecules regulated by certain compound. However, this method needs stable internal reference genes to analyze the expression change of the targets. In this study, the transcriptional stabilities of several traditional reference genes were evaluated in colon cancer cells treated with aspirin, and also, the suitable internal reference genes were screened by using a microarray and were further identified by using the geNorm and NormFinder softwares, and then were validated in more cell lines and xenografts. We have showed that three traditional internal reference genes, β-actin, GAPDH and α-tubulin, are not suitable for studying gene transcription in colon cancer cells treated with aspirin, and we have identified and validated TMEM208 and PQLC2 as the ideal internal reference genes for detecting the molecular targets of aspirin in colon cancer *in vitro* and *in vivo*. This study reveals stable internal reference genes for studying the target genes of aspirin in colon cancer, which will contribute to identify the molecular mechanism behind aspirin preventing colon cancer.

## INTRODUCTION

Aspirin, one of the most frequently used non-steroidal anti-inflammatory drugs (NSAIDs), has been demonstrated to possess an attractive effect on cancer chemoprevention [1–3]. The anti-cancer effect of aspirin was first identified in animal models in 1970s [4]. Subsequently, numerous epidemiological studies based on large scale population suggested that regular use of aspirin is significantly associated with lower risk of developing colorectal cancer [5–8], moreover, aspirin use not only reduced the incidence, but also reduced the mortality and metastasis of colon cancer [9, 10]. However, compared with its well-known chemoprevention effect, the molecular mechanism of aspirin preventing the tumorigenesis and progression of colon cancer is largely unknown. Thus identification of the target molecules of aspirin is an attractive topic in the cancer chemoprevention field.

One of the most frequently used strategies to identify the target molecules regulated by a certain compound is to determine the altered gene expression after treatment with that compound. Conventional experimental techniques used for evaluating gene expression levels include microarray analysis and quantitative reverse transcription polymerase chain reaction (qRT-PCR). Microarray is appropriate for detection of the global gene expression profiling [11, 12], whereas qRT-PCR is an efficient and sensitive method for measuring the expression of a limited number of genes [13, 14].To measure the expression change of certain genes in different experimental settings by using qRT-PCR, it is necessary to normalize the qRT-PCR data by referencing the expression of internal reference genes [15–18] that are usually house-keeping genes, such as β-actin, glyceraldehyde-3-phosphatedehydrogenase (GAPDH) and α-tubulin [19, 20], because of the fact that the expression of these genes is stable and is at a constant level under most experimental conditions [13, 21]. However, there are evidences suggesting that the transcript levels of some internal reference genes may be changed under certain conditions, such as drug treatment, serum stimulation and hypoxia [22, 23].

It has been reported that aspirin affects the cellular metabolism by targeting AMPK and mTOR [24, 25], which implies that the expression of metabolism associated gene GAPDH may be affected by aspirin. Additionally, it has also been suggested that the RNA molecules may be widely acetylated at the 2′-OH of the nucleotide, and this modification would impair the splicing, stability and translation of RNAs [26]. Therefore, whether aspirin treatment affects the transcriptional stability of the traditional internal reference genes is unknown. In this study the transcriptional stabilities of several traditional reference genes were evaluated in colon cancer cells treated with aspirin, also, the suitable internal reference genes were screened by using microarray assay and were further identified by using the geNorm and NormFinder softwares.

## RESULTS

### Aspirin alters the expression of traditional internal reference genes in colon cancer cells

**Figure 1:**
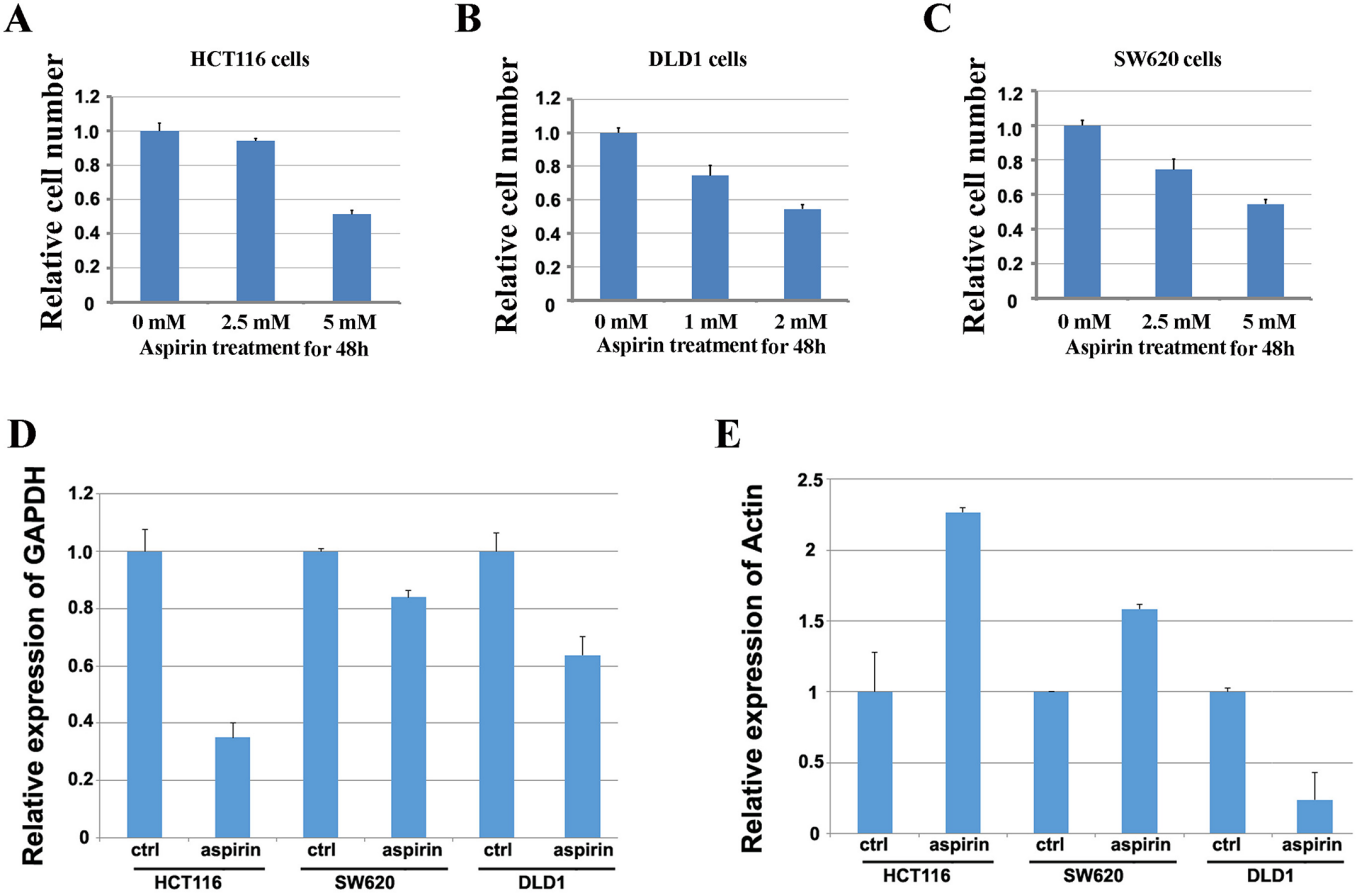
Screening the doses of aspirin to identify the reference genes in colon cancer cells treated with aspirin (**A**) Screening the dose of aspirin in HCT116 cells by detecting its inhibitory effect on cell proliferation. (**B**) Screening the dose of aspirin in DLD1 cells by detecting its inhibitory effect on cell proliferation. (**C**) Screening the dose of aspirin in SW620 cells by detecting its inhibitory effect on cell proliferation. (**D**) Detection of the expression of GAPDH in three colon cancer cell lines treated with aspirin. E. Detection of the expression of β-actin in three colon cancer cell lines treated with aspirin.

Since the plasma concentrations of intact acetylsalicylic acid, the primary metabolite of aspirin, is yielded up to 2.5 mM when aspirin is used to treat inflammatory diseases [27] and also the estimated concentration of aspirin in gastrointestinal mucosa epithelium where it is absorbed would be more higher than that, thus 1 mM to 15 mM is the most frequently used dose of aspirin in previous studies [24, 25]. Thus the concentrations of aspirin used in this study were optimized from 1 mM to 15 mM, and the effectiveness of aspirin was determined by cell growth suppression. By treating HCT116, DLD1 and SW620 cells with different concentrations of aspirin for 48h, we found that aspirin inhibited the proliferation of colon cancer cells in a dose dependent manner (Figure 1A–1C). The 50% inhibitory effect of aspirin on the proliferation of HCT116, SW620 and DLD1 cells is around at 5 mM, 5 mM and 3 mM respectively (Figure 1A–1C). Then we used these doses of aspirin to treat these three cell lines for further study.

To learn if aspirin treatment affects the expression of traditional internal reference genes, HCT116, DLD1 and SW620 cells were treated with and without aspirin for 24h respectively. Then, the expression change of *β*-actin or GAPDH was detected by using quantitative real-time PCR with *α*-tubulin as the internal reference gene. The results showed that expression of GAPDH decreased in three cell lines treated with aspirin (Figure 1D), whereas the expression of β-actin in HCT116 and SW620 cells treated with aspirin significantly increased, but decreased in aspirin treated DLD1 cells (Figure 1E). These results suggested that aspirin treatment altered the expression of traditional reference genes, thus it is necessary to identify the appropriate internal reference genes for studying the gene expressional alteration after aspirin treatment.

### Screening the candidate reference genes in colon cancer cells treated with aspirin by using microarray assay

**Figure 2:**
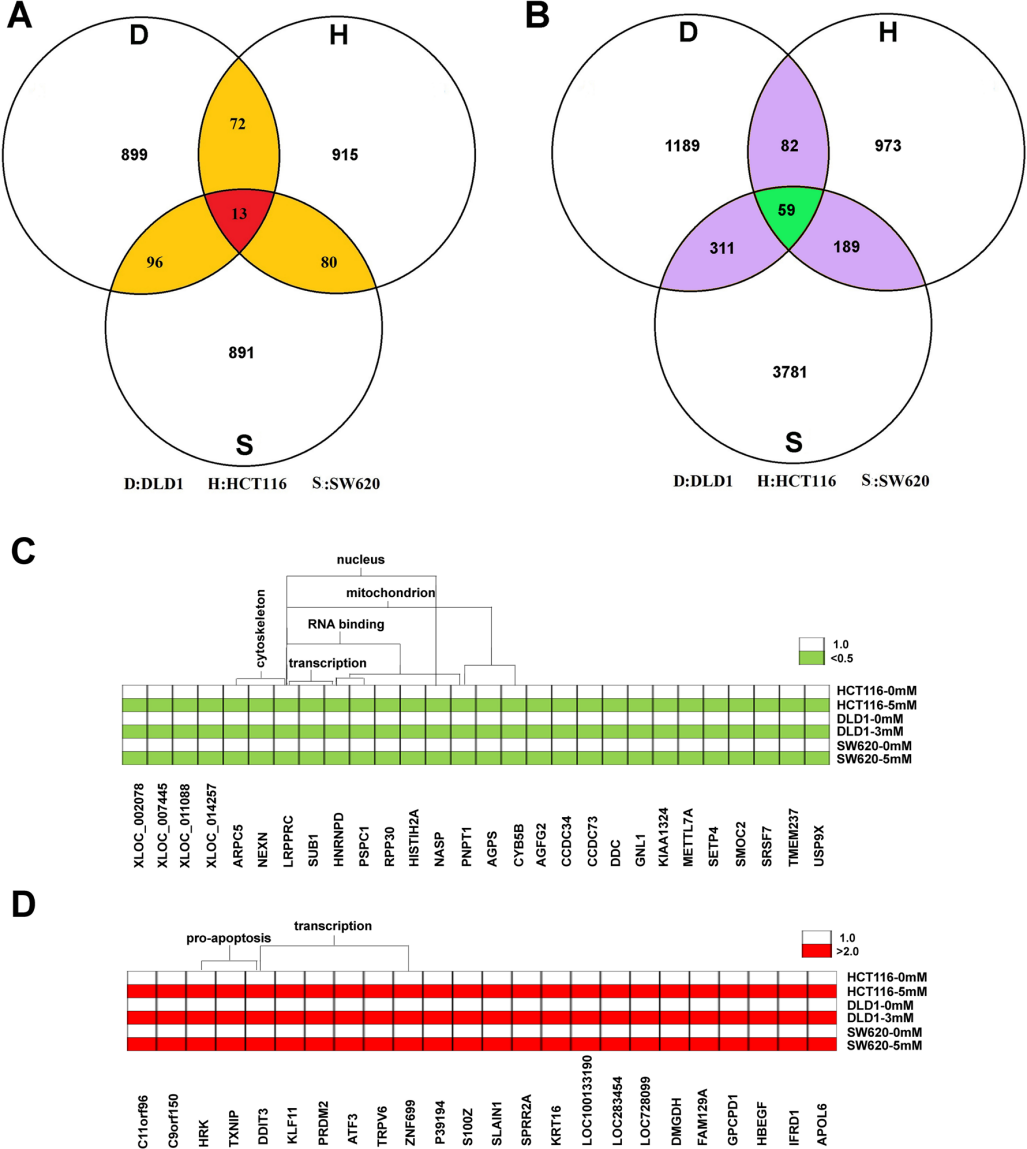
Screening candidate reference genes for normalizing mRNA expression in aspirin treated colon cancer cells (**A**) Thirteen stably expressed genes were screened as the candidates of the internal reference genes by using microarray. (**B**) There are totally 59 transcripts significantly changed in three cell lines after aspirin treatment. (**C**) The commonly down-regulated mRNA in three cell lines after aspirin treatment. (**D**) The commonly up-regulated mRNA in three cell lines after aspirin treatment.

To screen the candidate reference genes in colon cancer cells treated with aspirin, HCT116, SW620 and DLD1 cells were treated with 5 mM, 5 mM and 3 mM of aspirin for 24 h respectively, and then the microarray was employed to detect the global gene expression after aspirin treatment. The expression profiles of global genes for these three cell lines after aspirin treatment are shown in Supplementary Table 1. To screen the reference genes, we focused on the stably expressed genes as candidates, with a change fold less than 0.01 as cutoff to define stable expression in each cell line. There are about 1,100 candidate transcripts that meet this standard in each cell line. Finally, the intersections of independent candidates from three cell lines were considered as the candidate reference genes for further validation. Totally, we screened 13 reference gene candidates (Figure 2A), the information about these genes is listed in Table 1. Additionally, three traditional reference genes, GAPDH, *α*-tubulin and *β*-actin, were also considered to be evaluated together with these 13 candidates.

**Table 1:** The list of candidate internal reference genes

Number	Gene Symbol	Description	Chromosome
1	TMEM208	transmembrane protein 208	Chr16
2	GUSBP5	glucuronidase, beta pseudogene 5	Chr4
3	KRTAP1-3	keratin associated protein 1-3	Chr17
4	RPS25	ribosomal protein S25	Chr11
5	SLC18A3	solute carrier family 18 (vesicular acetylcholine), member 3	Chr10
6	MTDH	metadherin	Chr8
7	RPL36	ribosomal protein L36	Chr19
8	PQLC2	PQ loop repeat containing 2	Chr1
9	FAM208B	family with sequence similarity 208, member B	Chr10
10	NDST2	N-deacetylase/N-sulfotransferase (heparanglucosaminyl) 2	Chr10
11	IL17RC	interleukin 17 receptor C	Chr3
12	RPL8	ribosomal protein L8	Chr8
13	RPL23A	ribosomal protein L23a	Chr17

In addition to screening the candidates for reference genes, we also identified the potential target genes regulated by aspirin according to microarray data. We found that there were totally 59 transcripts significantly changed in three cell lines after aspirin treatment (Figure 2B). Especially, there are 32 transcripts representing 28 genes that were down-regulated; whereas there are 27 transcripts representing 24 genes that were up-regulated after aspirin treatment. The functional annotation clustering analysis (https://david-d.ncifcrf.gov/) suggested that aspirin significantly inhibited the expression of mitochondrion genes and cytoskeleton genes (*p* < 0.01) (Figure 2C); but significantly promoted the expression of pro-apoptosis genes (*p* < 0.01) (Figure 2D). Additionally, aspirin treatment also affects the expression of numerous transcription factors (*p* < 0.01) (Figure 2C and 2D).

### Identification of the stable internal reference genes in colon cancer cells treated with aspirin

The expression of these 16 candidate reference genes in three cell lines treated with aspirin was detected by Real-time PCR, and then the expression stability of these genes was evaluated by using geNorm and NormFinder softwares. GeNorm software automatically calculates the average expression stability value (M) for genes [11]. A lower *M* value means that the gene's expression is more stable and it is more suitable as an internal reference gene. The NormFinder software also determines the reference genes by calculating a value, and a lower value means that the gene is suitable as a reference gene. In order to get reliable results from RT-PCR research, it is suggested to combine and use two or more reference genes. The geNorm software also calculates the optimal number of reference genes combined to use for normalization based on a pair wise variation (Vn/(n+1)) analysis [22, 28].

**Figure 3:**
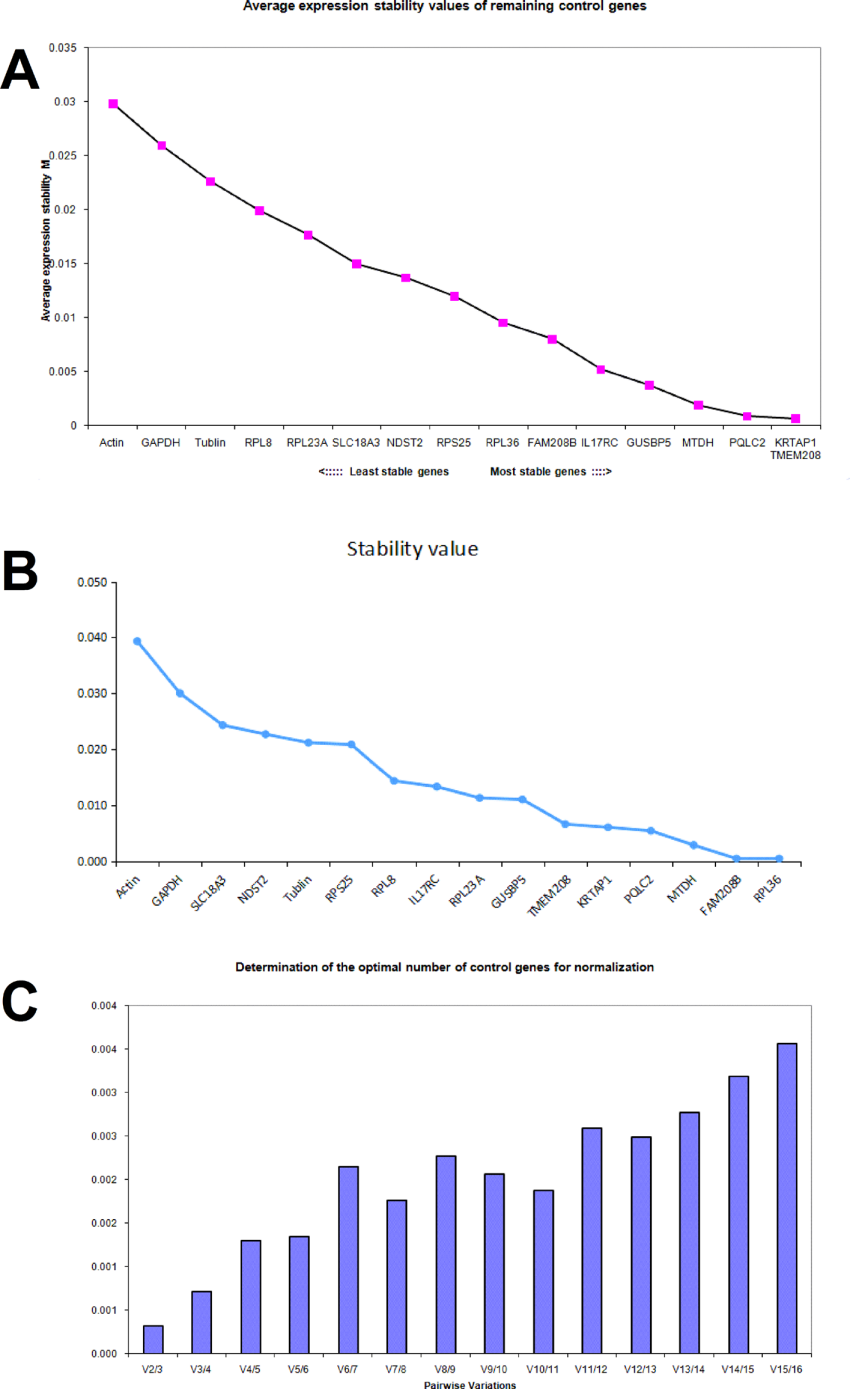
Identifying reference genes for normalizing mRNA expression in aspirin treated DLD1 cells (**A**) Identifying reference genes by using geNorm software. (**B**) Identifying reference genes by using NormFinder software. (**C**) Optimizing the number of reference genes in aspirin treated DLD1 cells.

In DLD1 cells treated with aspirin, the average *M* value of RPL36, FAM208B, IL17RC, GUSBP5, MTDH, PQLC2, KRTAP1 and TMEM208 calculated by geNorm was less than 0.01 respectively (Figure 3A), and these genes were considered to be stable in DLD1 cells treated with aspirin. Notably, three traditional internal reference genes β-actin, GAPDH and α-tubulin showed the highest average M value in aspirin treated DLD1 cells, which means that they are not suitable as internal reference genes; this result is consistent with microarray data (Figure 3A). The expression stability of these candidate genes in DLD1 cells was also analyzed by NormFinder software (Figure 3B). With 0.01 as the cutoff, MTDH, PQLC2, KRTAP1 and TMEM208 were considered for ideal internal reference genes. Thus the best internal reference genes in aspirin treated DLD1 cells are MTDH, TMEM208, PQLC2 and KRTAP1. Additionally, the geNorm analysis showed that the V2/3 value was dramatically lower in aspirin treated DLD1 cells (Figure 3C), which suggests that the optimal number of reference genes is two, thus the use of any two of these four genes in combination could be ideal internal reference gene in DLD1 cells treated with aspirin.

**Figure 4:**
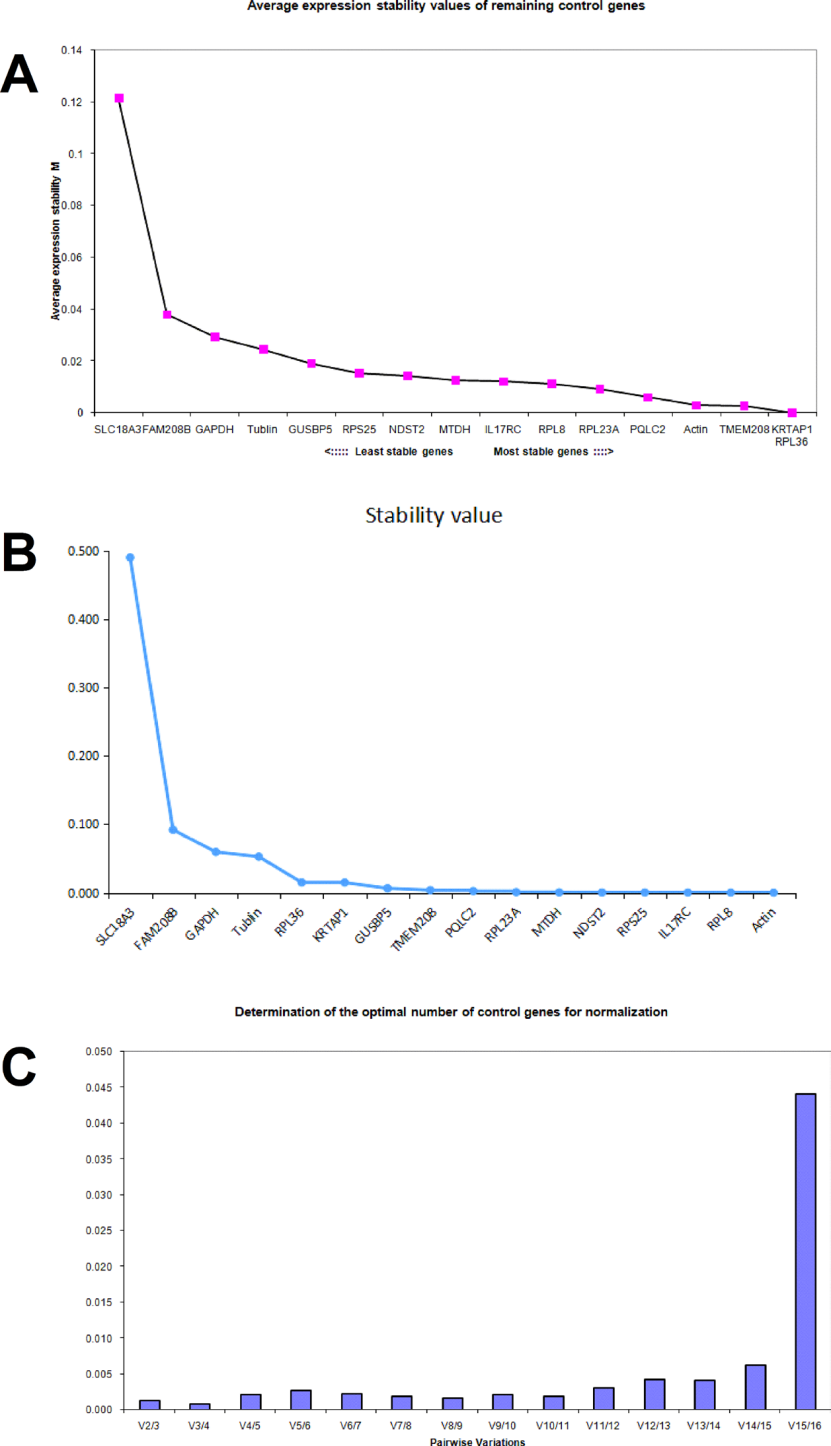
Identifying reference genes for normalizing mRNA expression in aspirin treated SW620 cells (**A**) Identifying reference genes by using geNorm software. (**B**) Identifying reference genes by using NormFinder software. (**C**) Optimizing the number of reference genes in aspirin treated SW620 cells.

In SW620 cells treated with aspirin, the geNorm average M value of each gene was calculated respectively and was shown in Figure 4A. The average *M* value of PQLC2, β-actin, TMEM208, KRTAP1 and RPL36 was less than 0.01, thus these five genes were considered to be stably expressed in SW620 cells treated with aspirin. Whereas the NormFinder analysis suggested that TMEM208, GUSBP5, PQLC2, RPL23A, MTDH, NDST2, RPS25, IL17RC and RPL8 were the ideal internal reference genes in SW620 cells, with 0.01 as the cutoff (Figure 4B). Thus the best references for normalization of gene expression in SW620 cells treated with aspirin are PQLC2 and TMEM208. Additionally, the geNorm analysis showed that the V2/3 value and V3/4 value was most lowest in aspirin treated SW620 cells (Figure 4C), which suggests that the optimal number of reference genes is two or three, thus the combination of PQLC2 and TMEM208 are the best internal reference genes in SW620 cells treated with aspirin.

**Figure 5:**
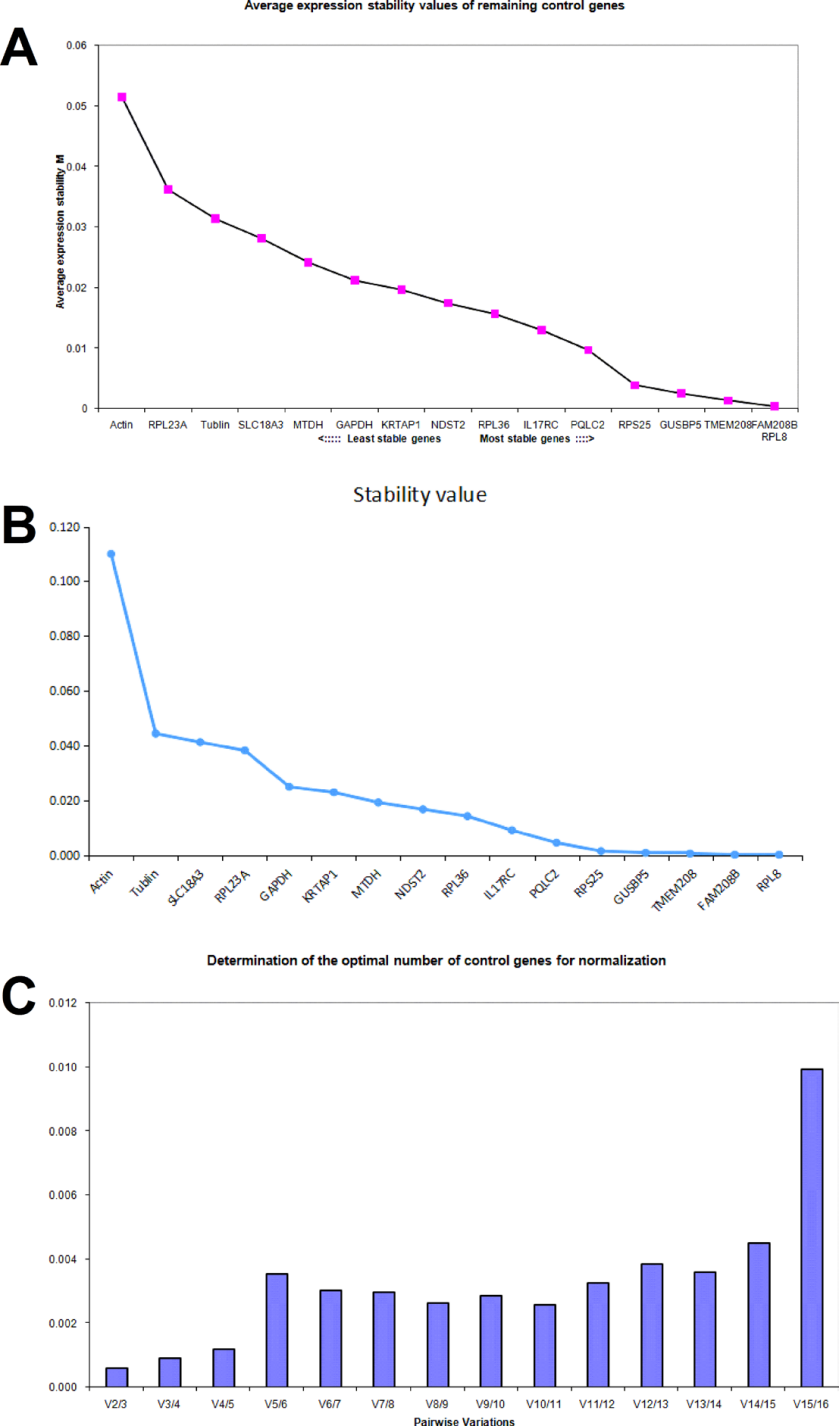
Identifying reference genes for normalizing mRNA expression in aspirin treated HCT116 cells (**A**) Identifying reference genes by using geNorm software. (**B**) Identifying reference genes by using NormFinder software. (**C**) Optimizing the number of reference genes in aspirin treated HCT116 cells.

In HCT116 cells treated with aspirin, the *M* value of PQLC2, RPS25, GUSBP5, TMEM208, RPL8 and FAM208B calculated by geNorm was less than 0.01 respectively (Figure 5A). Whereas the NormFinder analysis suggested that RPL36, PQLC2, FAM208, GUSBP5, IL17RC and TMEM208 were the ideal internal reference genes in HCT116 cells (Figure 5B). Thus the best references for normalization in HCT116 cells treated with aspirin are PQLC2, TMEM208, FAM208 and GUSBP5. Additionally, the geNorm analysis showed that the V2/3 value was the most lowest in aspirin treated HCT116 cells (Figure 5C), which suggests that it is ideal enough to combine and use two of these identified reference genes as the internal reference genes in HCT116 cells treated with aspirin.

The stable reference genes in DLD1, HCT116 and SW620 colon cancer cell lines treated with aspirin based on the geNorm and NormFinder analysis are summarized in Table 2. Taken together, we identified that TMEM208 and PQLC2 are the optimized internal reference genes in colon cancer cells treated with aspirin.

**Table 2:** Summary about the stable reference genes in three colon cancer cells

Cell lines	Stably expressed genes identified by geNorm	Stably expressed genes identified by NormFinder	Commonly identified reference genes
DLD1	RPL36, FAM208B, IL17RC, GUSBP5, MTDH, PQLC2, KRTAP1, TMEM208	MTDH, PQLC2, KRTAP1, TMEM208	MTDH, TMEM208, PQLC2, KRTAP1,
SW620	PQLC2, Actin, TMEM208, KRTAP1, RPL36	TMEM208, GUSBP5, PQLC2, RPL23A, MTDH, NDST2, RPS25, IL17RC, RPL8	TMEM208, PQLC2
HCT116	PQLC2, RPS25, GUSBP5, TMEM208, RPL8, FAM208B	RPL36, PQLC2, FAM208, GUSBP5, IL17RC, TMEM208	PQLC2, TMEM208, FAM208, GUSBP5

### Validation of TMEM208 and PQLC2 as the ideal internal references widely used in colon cancer treated with aspirin

**Figure 6:**
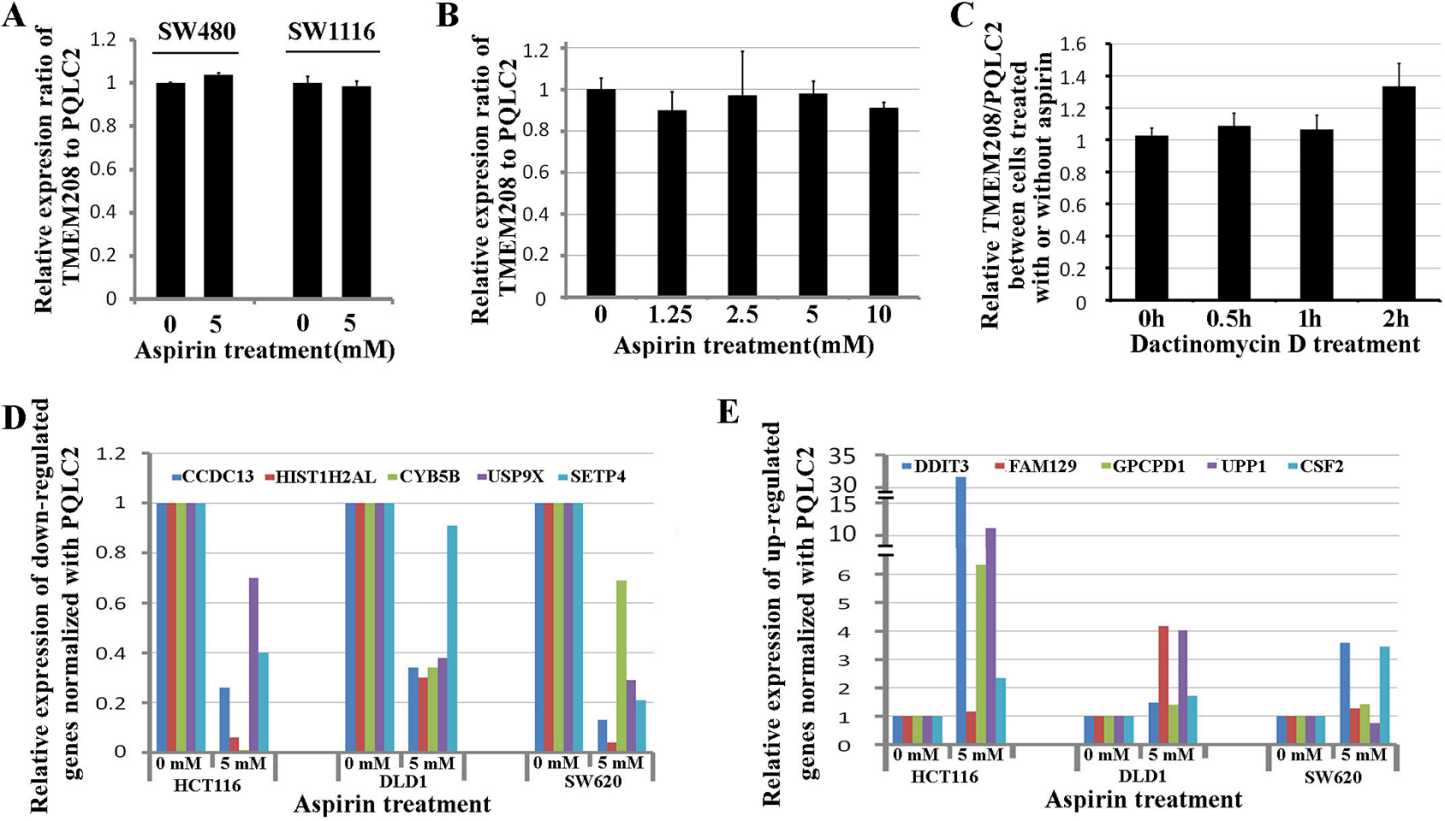
Validation of TMEM208 and PQLC2 as ideal reference genes in colon cancer cells (**A**) Validation of TMEM208 and PQLC2 as ideal reference genes in SW480 and SW1116 cells. (**B**) Changing the concentrations of aspirin does not alter the expression of TMEM208 and PQLC2. (**C**) aspirin treatment does not affect the mRNA decay of TMEM208 and PQLC2. (**D**) Validation of the down-regulated genes in three colon cancer cells treated with aspirin using PQLC2 as internal reference gene. (**E**) Validation of the up-regulated genes in three colon cancer cells treated with aspirin using PQLC2 as internal reference gene.

**Figure 7:**
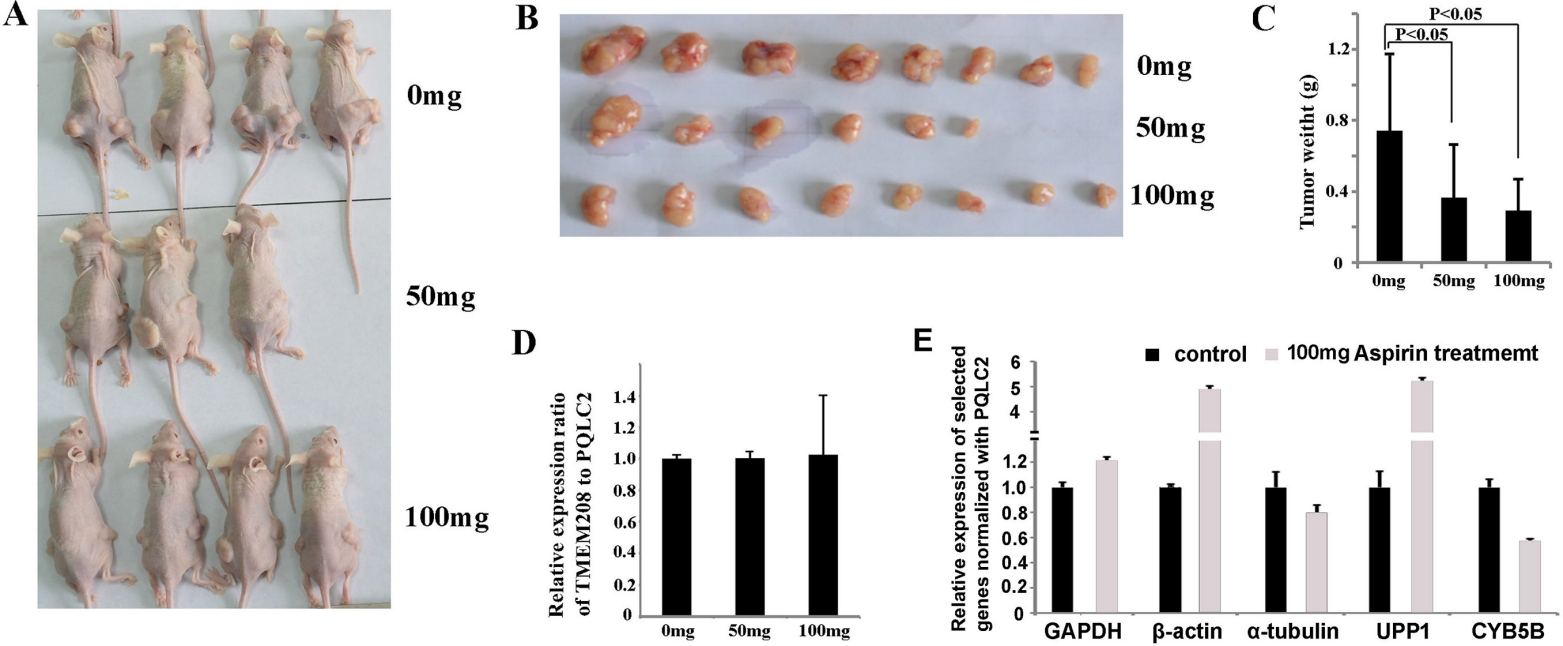
Validation of TMEM208 and PQLC2 as the ideal internal reference gene in xenograft of colon cancer treated with aspirin (**A**) The illustration of nude mice bearing colon cancer subcutaneously. (**B**) The illustration of tumors with and without aspirin treatment. (**C**) aspirin treatment significantly decreased the weight of tumor xenografts *in vivo* (*p* < 0.05). (**D**) The expression of TMEM208 and PQLC2 is not altered in the xenografts of colon cancer after aspirin treatment. (**E**) The relative expression of several selected genes (three traditional reference genes and two novel identified target genes of aspirin) in the xenografts treated with aspirin (100 mg) normalized with PQLC2.

To detect if TMEM208 and PQLC2 could be widely used as the ideal internal reference genes in colon cancer cells treated with aspirin, we additionally tested them in SW480 and SW1116 cells. Supposed that aspirin treatment causes the expression change in either TMEM208 or PQLC2, the ratio of TMEM208 to PQLC2 will be changed. Thus we detected the ratio of TMEM208 to PQLC2 to identify if they are stable after aspirin treatment. Our result showed that 5 mM aspirin treatment did not change the ratio of TMEM208 to PQLC2 in both SW480 and SW1116 cells (Figure 6A). Furthermore, we detected whether different concentrations of aspirin treatment caused the expression change in TMEM208 and PQLC2, and the result showed that the expression of both genes was stable in colon cancer cells treated with different concentrations of aspirin (Figure 6B). Moreover, we detected that if aspirin treatment affected the mRNA decay of TMEM208 and PQLC2 in colon cancer cells. HCT116 cells were treated with and without 5 mM aspirin for 24 h, and then further treated with 5 μM Dactinomycin D, an inhibitor of RNA polymerase, for 0 h, 0.5 h, 1 h and 2 h respectively [29–31]. Finally, the relative ratio of TMEM208 to PQLC2 in cells with aspirin and without aspirin treatment at different time points after Dactinomycin D treatment was calculated respectively, and the result showed that the relative ratio of TMEM208 to PQLC2 was identical in HCT116 cells with and without aspirin treatment at each time point after Dactinomycin D treatment (Figure 6C), which suggests that aspirin treatment does not affect the mRNA decay of TMEM208 and PQLC2. Additionally, we validated the expression change of microarray data with our identified internal reference genes. Most of the commonly up- and down-regulated genes after aspirin treatment in the three cell lines were validated by using PQLC2 as internal reference gene (Figure 6D and 6E). Finally, we evaluated that if TMEM208 and PQLC2 are ideal to serve as internal references for colon cancer treated with aspirin *in vivo*. After aspirin treatment for one month, the tumor weight was significantly decreased in a dose dependent manner (Figure 7A–7C), whereas the ratio of TMEM208 to PQLC2 was not altered in the xenografts of colon cancer after aspirin treatment (Figure 7D). By contrast, the expression of traditional reference genes (GAPDH, α-tubulin and β-actin) in the xenografts treated with aspirin was significantly changed (Figure 7E). Furthermore, we evaluated the expression of several target genes that have been identified by microarray in the xenografts after aspirin treatment with PQLC2 as the internal reference gene, and we demonstrated that the expression change of detected genes after aspirin treatment in the xenografts was consistence with that *in vitro* (Figure 7E). These results suggested that TMEM208 and PQLC2 were ideal internal reference genes for normalizing mRNA expression in colorectal cancer treated with aspirin.

## DISCUSSION

Gene transcription analysis is widely used for molecular biological research and is critical for understanding biological processes and identifying gene function [32]. It is generally accepted that measuring the alteration of gene expression requires internal control genes and those ideal control genes should be stably expressed under all experimental conditions. In most experimental conditions, the transcription of β-actin, GAPDH and α-tubulin are stable, thus these three genes are usually used as the internal reference genes. However, their transcription may be changed in certain conditions. For example, the expression of β-actin and GAPDH are significantly elevated corresponding to serum stimulation; whereas the expression of GAPDH is significantly induced under hypoxia. Thus it is necessary to identify stable internal reference genes in different experiment settings. Recently, it is reported that B2M and 18S RNA are suitable internal reference genes in serum stimulated samples [19]; GUSB and PPIA are believed to serve as internal reference genes in ovarian cancer [33], while RPL13A is identified to stably transcript in ovarian cancer cells treated with PTX and HCPT [28].

In this study, we have demonstrated that *β*-actin, GAPDH and *α*-tubulin are not suitable as internal reference genes in colon cancer cells treated with aspirin. Therefore, we have performed a set of gene expression microarray to screen the candidate internal reference genes in human colon cancer cell lines (DLD1, SW620 and HCT116) treated with aspirin. Subsequently, we choose 13 candidate genes from microarray data together with *β*-actin, GAPDH and *α*-tubulin for further identification by using geNorm and NormFinder algorithms. The geNorm algorithm is developed by Jo Vandesompele et al. in 2002. This measure is based on the principle that the expression ratio of two ideal control genes is identical in all the experimental conditions. If the ratios of two reference genes is less varied in all samples, which means that the candidate gene is constantly expressed and could be served as internal reference gene [11]. NormFinder is another most frequently used strategy to normalize quantitative RT-PCR data developed by Andersen et al. in 2004. They developed a mathematical model to estimate the expression variation of each evaluated candidate gene by calculating the log-transformed measured gene expression for candidate gene in a particular sample of certain group in order to estimate the intra-group and intergroup variation. The estimated intergroup expression variations allow for selection of the suited genes, whereas the intra-group expression variations suggest the number of genes needed. This strategy takes into account systematic differences between sample subgroups and thus provides more directly and robustly evaluation of gene expression stability [15]. Our results show that there are two genes, namely TMEM208 and PQLC2, which remain transcriptionally stable in colon cancer treated with aspirin. Thus TMEM208 and PQLC2 are the appropriate reference genes for gene transcription analysis in colorectal cancer cells following aspirin treatment. TMEM208 gene encodes a highly conserved protein that is localized in the endoplasmic reticulum (ER) and regulates autophagy and ER stress [34]. PQLC2 gene encodes a lysosomal cationic amino acid transporter that maintains amino acid homeostasis [35].

Additionally, by microarray analysis, we identified several novel target genes regulated by aspirin in colon cancer cells. Totally, we found 28 down-regulated and 24 up-regulated genes corresponding to aspirin treatment in three colon cancer cell lines. Among the down-regulated genes, there are four mitochondria functional genes including LRPPRC, PNPT1, AGPS and CYB5B. Mitochondria associated proteins are involved in the regulation of cellular apoptosis and metabolism. For example, LRPPRC is found to be highly expressed and subsequently prevent the apoptosis or induce autophagy in several cancer types [36, 37]. Thus inhibiting the expression of these mitochondria associated genes may partly contribute to understand the mechanisms about aspirin promoting the apoptosis and inhibiting cellular metabolism in many cancer cells. Additionally, aspirin treatment also significantly decreased the expression of genes encoding cytoskeleton including ARPC5, NEXN and LRPPRC, which may be helpful to understand the mechanisms behind aspirin suppressing the metastasis of cancer cells. Whereas among the up-regulated genes, there are three pro-apoptosis genes including HRK, TXNIP and DDIT3, which may also helpful to explain how aspirin can induce cellular apoptosis in colon cancer.

In conclusion, we have not only found that three traditional internal reference genes are not suitable for studying gene transcription in colon cancer cells treated with aspirin, but also identified TMEM208 and PQLC2 as the ideal internal reference genes in detecting the molecular targets of aspirin in colon cancer cells. Additionally, this study supports the idea that genes do not transcribe stably in all types of cancer, and it is necessary to select the suitable reference gene to analyze the transcription profile of cancer cells receiving different treatments.

## MATERIALS AND METHODS

### Cell culture and aspirin treatment

The human colorectal cancer cell lines HCT116, SW620, SW480, SW1116 and DLD1 were cultured in DMEM (Hyclone, Logan, UT, USA) containing 10% fetal bovine serum (FBS; Invitrogen, Carlsbad, CA, USA) with 100 IU/ml penicillin and 100 μg/ml streptomycin at 37°C in a 5% CO_2_ humidified atmosphere. Aspirin was purchased from Sigma (Sigma-Aldrich, St. Louis, MO, USA), and dissolved in ethanol to prepare the 1 M storage solution. The inhibitor of RNA polymerase Dactinomycin D was purchased from Sigma and dissolved in DMSO to prepare the 1 mg/ml storage solution.

### Total RNA extraction and cDNA synthesis

Total RNA was extracted from cultured cells with TRIzol reagent (Invitrogen, Life Technologies). The RNA concentration was measured by A260/A280 and A260/A230 absorbance ratios with NanoDro2000 (Beckman Coulter, Brea, CA, USA). A total of 1 μg RNA was reverse transcribed into cDNA by Transcript All-in-one Fisrt-Strand cDNA Synthesis SuperMix for qPCR (TransGen Biotech, Beijing, China) by incubating at 42°C for 15 min, followed by 85°C for 5 s.

### Microarray hybridization and data pre-processing

Briefly, total RNA was isolated using TRIzol Reagent following the manufacturer's instructions and checked for a RIN number to inspect RNA integration by an Agilent Bioanalyzer 2100 (Agilent technologies, Santa Clara, CA, US). Qualified total RNA was further purified, amplified and labeled by Low Input Quick Amp Labeling Kit (Aglient technologies, Santa Clara, CA, US) following the manufacturer's instructions. Labeled cRNA were purified and used as templates for reverse transcriptional labeling of fluorescent cDNA probes. Each Slide was hybridized with 1.65 ug Cy3-labeled cDNA using Gene Expression Hybridization Kit (Aglient technologies) in Hybridization Oven (Aglient technologies), according to the manufacturer's instructions. After 17 hours hybridization, slides were washed and scanned by Agilent Microarray Scanner (Aglient technologies) and the images were processed using the Agilent Feature Extraction v.11 software (Agilent Technologies). Quality Control report was used to evaluate the reproducibility and reliability of each microarray. The GeneSpring v.13.1 software (Agilent Technologies) was used to perform data analysis. Especially, the 75th percentile signal value was used to normalize data and the *T*-test was used to detect the difference of gene expression [38].

### Real-time qRT-PCR assays

The relative expression of genes was detected by using the SYBR Green PCR Mix (BIORESEARCHER, Beijing, China) by Bio-Rad CFX96TM Real-Time System. The PCR assays were performed at 95°C for 5 min and then 40 cycles of denaturation at 95°C for 10 s and amplification at 60°C for 30 s, followed by melting curve analysis by progressive heating from 65 to 95°C. Primer sequences are summarized in Table 3.

**Table 3:** Primers used in this study

Gene Name	Primers(5′-3′)	Product(bp)
RPS25	F:CGCCTAAGGACGACAAGA	112
	R:TTGCCTTTGGACCACTTC	
GUSBP5	F:GGGATGCTGTACCCAAGA	126
	R:CAGACGCCGATACCACTA	
SLC18A3	F:GAGCCACCGCAAGGTCTGT	107
	R:TCCTCGCACGCATCAAAA	
MTDH	F:AAACGTGATAAGGTGCTG	105
	R:AAATGATGCGGTTGTAAG	
RPL36	F:TCGTGCGGGACATGATTC	150
	R:CCCCACCCTTTTCTTGATA	
KRTAP1-3	F:GCGGATTTCCTAGCTTCTCA	150
	R:CCACCTGATACGGGTGCT	
FAM208B	F:TATCCTTCCGTGATCCTA	137
	R:ATTCTGGTCTTCTTGGGT	
TMEM208	F:GTCTGGTCCTTCTGGCTTCT	122
	R:TGCCGTTTCTCATTGTGC	
RPL23A	F:CCAACAAGCACCAGATTA	117
	R:CAGGAGCCAGTCGAACAT	
RPL8	F:GCACGGCTACATCAAGGG	130
	R:CTCGGCGGCAATGAACAG	
IL17RC	F:GGAGCCGTCGTGAACTGA	143
	R:CCCATCCTGTAGCCACTCG	
NDST2	F:TTGACCGCTACATCTTGG	108
	R:TGAGTTTGTTCTGGGTGG	
PQLC2	F:GCCTCAGATCCGCACCAA	104
	R:GCACGCTCAGCCCATACA	
GAPDH	F:GACCTGACCTGCCGTCTA	148
	R:AGGAGTGGGTGTCGCTGT	
ACTB	F:GGAAATCGTGCGTGACATT	113
	R:CAGGCAGCTCGTAGCTCTT	
TUBA1A	F:CTTGGAACCCACAGTCATT	112
	R:CCCTCGGGCATAGTTATT	

### Animal experiment

SW1116 cells were subcutaneously inoculated in the nude mice to evaluate the impact of aspirin on the expressional stability of internal reference genes. Briefly, eleven male nude mice were inoculated subcutaneously with SW1116 cells at both sides of the axillary fossa near the back, and each side was injected 1 × 10^6^ cells. One week after the inoculation, mice were randomly divided into three groups and then intragastric administration with 0 mg (*n* = 4), 50 mg (*n* = 3) and 100 mg (*n* = 4) of aspirin daily for one month. Then the mice were slaughtered and the tumors were weighted. Total RNA of each tumor was extracted using TRIzol reagent, and then the same amount of RNA from the tumors of the same treatment group was mixed together and used for detecting the impact of aspirin on the expression of selected genes.

### Data analyses

Two analysis softwares, geNorm (https://genorm.cmgg.be/) and NormFinder V19 (http://moma.dk/normfindersoftware; Department of Molecular Medicine, Aarhus, Denmark), were used to identify the stable reference genes. The data were presented as the mean ± SD. Comparisons between the groups were analyzed by Chi-square test, Student's *t* test or One-Way ANOVA analysis. *P* < 0.05 was defined as statistically significance.

## SUPPLEMENTARY MATERIALS FIGURES AND TABLES




