# Supplementary material for: Identification of TMEM208 and PQLC2 as reference genes for normalizing mRNA expression in colorectal cancer treated with aspirin
Source: Oncotarget. 2017 Feb 8;8(14):22759–71. doi: 10.18632/oncotarget.15191 (PMC5410260; doi:10.18632/oncotarget.15191)
Supplement: Supplementary file 1 [file oncotarget-08-22759-s001.pdf]

Identification of TMEM208 and PQLC2 as reference genes for normalizing mRNA expression in colorectal cancer treated with aspirin

Supplementary Materials

Supplementary Table 1: The expression profiles of global genes in three CRC cell lines after aspirin treatment.
See Supplementary_Table_1
